# Influence of Exposure to a Wet Atmosphere on the UV-Sensing Characteristics of ZnO Nanorod Arrays

**DOI:** 10.3390/ma17051053

**Published:** 2024-02-25

**Authors:** Maria Evstafieva, Arcady Redkin, Dmitry Roshchupkin, Tatyana Rudneva, Eugene E. Yakimov

**Affiliations:** Institute of Microelectronics Technology RAS, 6 Academician Ossipyan Str., 142432 Chernogolovka, Russiarochtch@iptm.ru (D.R.); ruta@iptm.ru (T.R.); yak@iptm.ru (E.E.Y.)

**Keywords:** ZnO nanorod array, CVD method, UV sensor, photoresponse, sensing properties, surface effects

## Abstract

Zinc oxide is a promising material for the creation of various types of sensors, in particular UV detectors. In this work, arrays of ordered nanorods were grown by chemical vapor deposition. The effect of environmental humidity on the sensing properties of zinc oxide nanorod arrays was investigated, and a prototype UV sensor using indium as an ohmic contact was developed. UV photoresponses were measured for the samples stored in dry and wet atmospheres. The increase in sensitivity and response of the ZnO nanorod arrays was observed after prolonged exposure to a wet atmosphere. A model was proposed to explain this effect. This is due to the formation of hydroxyl groups on the surface of zinc oxide nanorods, which is confirmed by FTIR spectroscopy data. For the first time, it has been shown that after storage in a wet atmosphere, the sensory properties of the structure remain stable regardless of the ambient humidity.

## 1. Introduction

Zinc oxide, an important semiconductor material, has a wide range of applications for UV devices [1,2,3,4] due to its high excitonic binding energy of ~60 meV and the large band gap of 3.37 eV at room temperature [5,6]. UV sensors based on wide-band one-dimensional oxide semiconductor materials have a number of advantages over other types of sensors, such as low cost, high sensitivity, high temperature resistance, radiation resistance, parameter stability, etc. Compared to film materials, arrays of one-dimensional nanostructures have a larger working surface area and therefore, a larger area of the adsorbing layer, high selectivity, and fast response. There are a large number of publications devoted to the synthesis of one-dimensional zinc oxide nanostructures. A number of publications report the synthesis of 1D ZnO nanostructures (e.g., nanorods) oriented perpendicular to the substrate in a more or less disordered spatial arrangement. In general, all methods for the synthesis and growth of ZnO nanostructures can be divided into gas phase growth and solution growth. The gas phase growth methods include carbo-thermal evaporation [7], magnetron evaporation [8], chemical vapor deposition (CVD) [9,10], pulsed laser deposition (PLD) [11,12], molecular beam epitaxy (MBE) [13], and metal organic vapor phase epitaxy (MOVPE) [14]. The main solution methods are the hydrothermal method [15], electrochemical deposition [16] and chemical bath deposition [17]. Compared to other methods, CVD provides high productivity, repeatability and excellent quality of the resulting material with comparative simplicity and accessibility.

One of the most important requirements for the operation of any industrial device is stable operation and resistance to environmental influences, in particular, atmospheric humidity. Research into the effects of humidity is not new. These are the necessary steps to assess the feasibility of the industrial production of devices. It is important to understand the degradation of the physical properties of structures under real atmospheric conditions. Zinc oxide is a material resistant to aggressive conditions. However, zinc oxide has poor solubility in water, which can be critical in the case of nanoscale structures. This is particularly relevant for sensors based on ZnO nanorods with developed surfaces. In this case, the surface effects of the nanorods play a very important role. It is known from the literature that ZnO is an n-type semiconductor due to the presence of intrinsic defects, such as oxygen vacancies [18,19]. According to the generally accepted mechanism, the processes of adsorption and desorption of gas molecules on the surface are responsible for the change in the electrophysical properties of ZnO nanorods under UV light irradiation [20]. Surface defects can also cause the adsorption of molecules, such as oxygen and water, which act as surface traps for free electrons in nanowires. The most significant role is played by surface effects associated with adsorbed oxygen molecules [21,22,23]. Another adsorbed component is water vapor, which is present in large quantities in the environment. The adsorption of water molecules on the surface has not yet been sufficiently studied due to the complex mechanism of water interaction with different planes of ZnO. Water is affected by chemical bonding, van der Waals forces, and hydrogen bonding, which can lead to partial or complete dissociation at defects [24,25]. The (1010) plane is the most energetically favorable for water adsorption. It has been shown in a number of works [24,25,26,27,28] that water forms a (2 × 1) superstructure with long-range order that exists up to temperatures close to the boiling temperature of water, which is also confirmed by computer modelling of this process. This superstructure thus consists of two water molecules, one of which is dissociated. In addition, adsorbed water molecules can form either a monolayer or multiple layers.

Surface effects associated with adsorbed oxygen and water molecules affect UV photosensitivity. As a result of exposure to a wet atmosphere, the sensing characteristics of the ZnO nanowire arrays change. A number of works have been devoted to the study of the effect of humidity on sensing characteristics [21,29,30,31]. However, the research results are rather controversial, and the effects are not sufficiently studied. In addition, the effect of adsorbed water on the surface also depends on the methods of obtaining the zinc oxide nanowires. For example, solution methods initially assume the presence of some water on the surface of the nanowires.

In this work, arrays of ordered nanorods were grown by the self-catalytic chemical vapor deposition method using the vapor-liquid-crystal mechanism. The main advantage of our method, described in our previous works [32,33], is the possibility of a controlled process, which makes it possible to control the main characteristics of the resulting one-dimensional nanocrystals: reproducibility, size, orientation relative to the substrate, and even shape. The effect of prolonged storage of the samples in a wet atmosphere on photosensitivity, changes in dark current (I_dark_), current under UV irradiation (I_UV_), and the I_UV_/I_dark_ response was studied. The time-dependent photoresponse of the ZnO nanorod array based photodetector to UV irradiation at room temperature in dry and wet atmospheres was investigated. The aim of our work was to study the influence of humidity on the sensory properties of the samples as a function of storage time.

## 2. Materials and Methods

Arrays of ordered ZnO nanorods were grown by the CVD method. The synthesis of zinc oxide nanocrystal arrays was carried out according to the following procedure. An alumina boat with granulated high-purity zinc (99.99%) was placed in the sealed end of an ampoule, with a wide slit on the open side on the top wall of the ampoule, opposite to which the substrates were placed with the working side up. The ampoule was placed in a horizontal quartz reactor with two heating zones, so that the zinc was in the evaporation zone and the substrates were placed in the growth zone. The substrates used were silicon {001}, quartz and glass. The synthesis was carried out at a reduced pressure of 10^3^ Pa in an inert gas stream (99.999% argon (Linde gas, Balashikha, Russia)) at a flow rate of 4 L/h. Oxygen (99.999%, Linde gas, Balashikha, Russia) was injected at 0.4 L/h. The temperatures in the evaporation and growth zones were 610 °C and 550 °C, respectively. The process was carried out according to the vapor-liquid-crystal mechanism. During the process, zinc vapors entered a higher temperature zone, from which zinc vapors with an argon stream entered the growth zone, where they partially condensed, forming an array of zinc nanodroplets of fairly uniform size on the substrates. This process is self-catalytic, i.e., the Zn nanodroplets act as catalysts. When oxygen reacts with liquid zinc, the produced oxide dissolves in the droplets, eventually forming a supersaturated solution from which solid ZnO crystallizes at the metal/substrate interface. This reaction results in the oriented growth of ZnO nanocrystals under the droplets. The synthesis was carried out for 30 min at a zinc flow rate of 15 g/h.

To study the sensory properties, the samples were annealed at 550 °C in air for 3 h. The temperature and annealing time were chosen from our previous experiments. The detailed effect of annealing on the electrical conductivity of zinc oxide nanorods has been described in the literature [34]. As shown in [35], this is sufficient to relax internal stresses and anneal defects. It is also technologically simpler than annealing in a vacuum or inert atmosphere. At higher temperatures, oxygen vacancies are created, and the crystal structure of the sample begins to shrink due to a change in morphology [36]. In [37], it was shown that subsequent annealing can significantly improve the properties of photodetectors. It has also been shown previously that annealing in air reduces both the dark current and the irradiation current by 3 orders of magnitude [38]. However, the reduction in dark current is much greater, resulting in a 2–3-fold increase in UV sensitivity.

A JSM 6490 scanning electron microscope (SEM) (JEOL LTD, Akishima, Tokio, Japan) equipped with the MonoCL3 cathodoluminescence system was used to analyze the morphology of the samples (beam energy 10 to 30 kV, maximum current 2 nA) and to record cathodoluminescence spectra. CL measurements were performed with the same excitation 10 kV and current 1 nA and the same detection system parameters with a resolution of less than 2 nm.

The crystallinity of an array of ZnO nanorods was studied by X-ray diffraction using a double-crystal X-ray diffractometer on a BRUKER D8 Discover (BRUKER, Berlin, Germany) laboratory X-ray source with a rotating copper anode (radiation CuKα1 = 1.54 Å). A Bruker Sentera micro-RAMAN microscope (BRUKER, Berlin, Germany) under excitation by a solid-state laser with a wavelength of 532 nm was used to record the Raman spectra of the samples. The Raman spectra of the samples were recorded using a Bruker Sentera micro-RAMAN microscope excited by a solid-state laser at a wavelength of 532 nm. Fourier transform infrared (FTIR) spectra were recorded using a Bruker Vertex 70 V spectrometer (BRUKER, Berlin, Germany) in the 400–4000 cm^−1^ range with a resolution of 4 cm^−1^. Nanorods were mechanically removed from samples stored in dry and wet atmospheres, then mixed with KBr (~1 mg: 300 mg) and pressed into pellets for IR absorption analysis.

To measure the electrical properties of the ZnO nanorod array, indium pressure contacts were used at a distance of 7 mm. According to the literature, indium forms ohmic contacts with zinc oxide [39], which was confirmed by linear voltammetric characteristics. This structure is a polycrystalline film (sublayer) on which UV-sensitive nanorods are arranged vertically, so that measured current between the contacts passes through the polycrystalline ZnO film in the lateral direction. A voltage of 10 V was applied to the sample using a power supply (APS-1333 from AKTAKOM (Eliks, Moscow, Russia)), with an output voltage setting error of 1%. The current was measured using a multimeter (AM 1109 from AKTAKOM (Eliks, Moscow, Russia)) and recorded by a computer program every 5 s. The error of the DC current measurement was 0.1%. In order to study the rise and fall times, the time dependence of the photoresponse was recorded when the UV radiation was switched on and off. The source of UV irradiation was a LUF-4 mercury fluorescent lamp with a maximum radiation of about 370 nm; the light flux density at a distance of 10 cm was 4 W/m^2^. The on and off times of the UV irradiation were 10 min. The response time and relaxation time were determined as the time to reach the current value equal to 90% of the limit value [40]. The photosensitivity to UV light was defined as (I_UV_ + I_dark_)/I_dark_ [21].

To investigate the effect of a wet atmosphere on the sensing properties of an array of ZnO nanorods, several pairs of samples were obtained simultaneously. One sample from each pair was placed in a desiccator with silica gel to create a dry atmosphere, and another sample was placed in a desiccator with an open container of water. The relative humidity in the second desiccator was close to 100%. The samples were kept in the desiccators for 25 days. At the same time, the samples were removed daily from the desiccators, and their time-dependent photoresponses were measured when the UV irradiation was switched on and off according to the method described above. After 25 days of storage, the samples were exchanged, and their UV photoresponses were measured for another 10 days.

## 3. Results and Discussion

### 3.1. ZnO Nanorods Arrays Characterization

**Figure 1:**
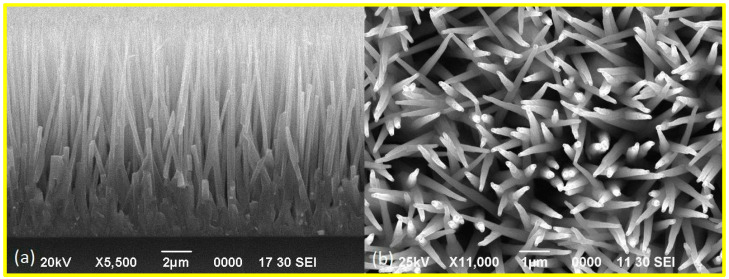
Typical SEM images of a 10 μm length ZnO nanorod array: (**a**) cross-sectional view, (**b**) planar view.

Figure 1 shows typical SEM images of the ZnO nanorod arrays obtained. The samples chosen for the study were polycrystalline films with vertically oriented nanorods on the surface of a glass substrate. The film thickness was approximately 4–5 μm, and the height of the nanorods was about 8–10 μm. Individual nanorods had an average diameter of about 0.2 µm. According to scanning electron microscopy data, the density of nanorods in the array was about 4 ×·10^8^/cm^2^. This density of nanorods can increase the effective working surface area by up to 20 times compared to planar or bulk materials, while having greater physical stiffness and good surface bonding compared to single nanowires.

**Figure 2:**
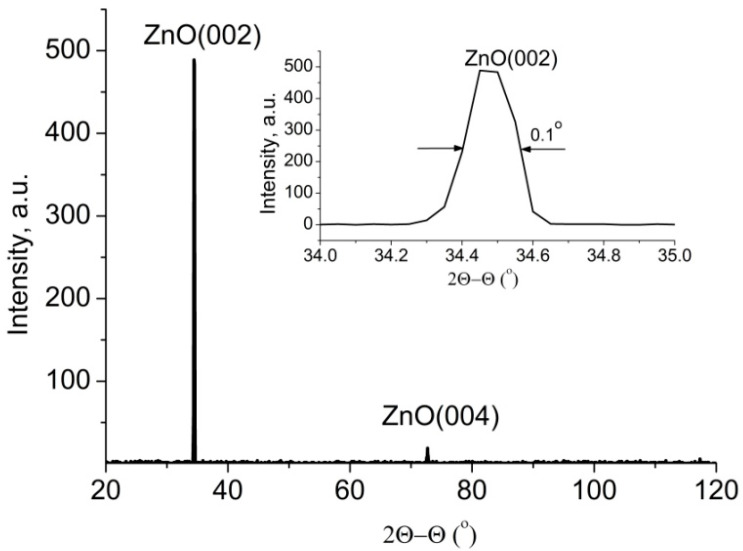
XRD spectrum of a ZnO nanorod array grown on a glass substrate. The inset shows the reflection peak (002) magnified.

The X-ray diffraction spectrum of ordered arrays of ZnO nanorods (Figure 2), shows only reflexes (002) and (004), which clearly illustrating the *c*-axis oriented growth.

The full width at half maximum (FWHM) of these peaks for ZnO nanorod arrays is 0.1–0.12° (inset of Figure 2). This indicates that the nanorods have a good crystalline structure and are well oriented.

**Figure 3:**
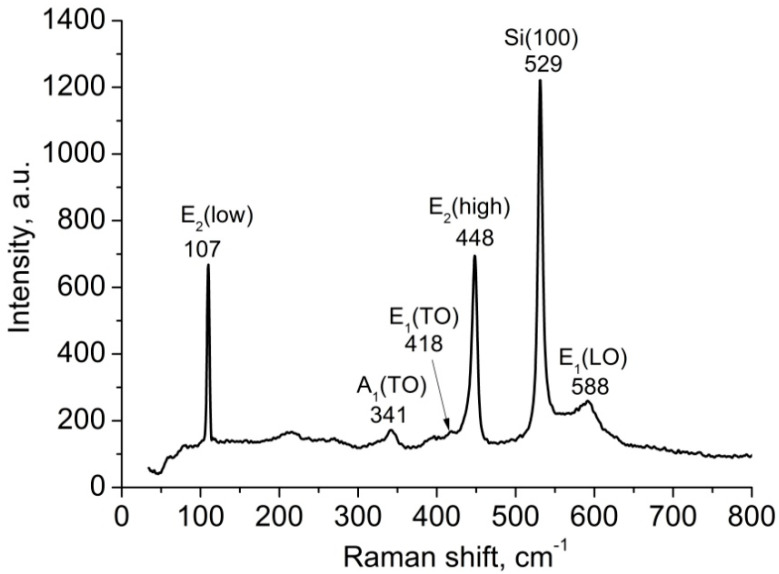
Raman spectrum of ordered ZnO nanorods array on a Si (100) substrate.

In the Raman spectrum of the obtained samples (Figure 3), the main intense peaks of E_2_(low) and E_2_(high) are observed at 107 and 448 cm^−1^, respectively. In the literature, the low-frequency mode E_2_(low) is most often associated with non-polar vibrations of the heavier sublattice of Zn atoms, while the high-frequency mode E_2_(high) is associated with the displacement of lighter oxygen atoms [41]. The half-widths and positions of these peaks are comparable to those found in bulk ZnO single crystals. The A_1_ and E_1_ modes are polar, and split into longitudinal (LO) and transverse (TO) optical components. The peaks that are assigned to the A_1_(TO), E_1_(TO), and E_1_(LO) modes were observed at 341, 418, and 588 cm^−1^, respectively.

**Figure 4:**
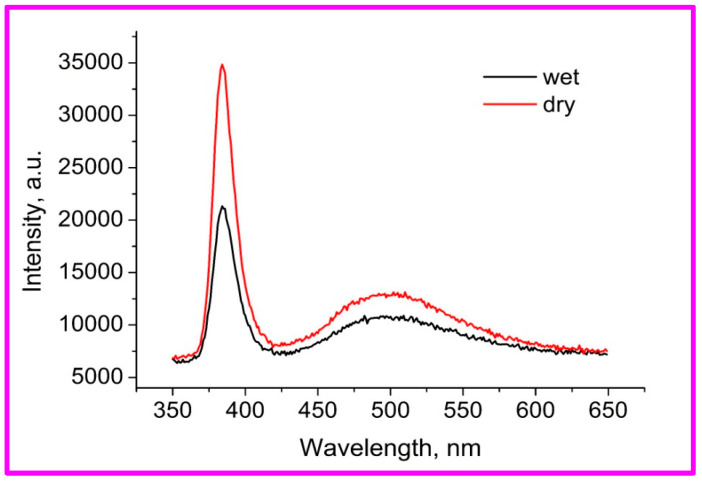
CL spectrum of ZnO nanorod arrays stored in dry and wet atmospheres for 25 days.

Figure 4 shows the room-temperature CL emission spectra of ZnO nanorod arrays stored in wet and dry atmospheres. The spectra contain narrow UV exciton peaks in the short-wavelength part of the spectrum with a maximum around 384 nm and a broad green emission line with a maximum around 500 nm in the visible region. The green emission band is usually associated with oxygen vacancies [42]. The oxygen vacancies also have a blue emission line at about 420 nm, but they are not observed in these cases. The relationship between these peaks provides a qualitative estimate of crystallinity. As can be seen from this figure, the sample in a wet atmosphere has a broader exciton peak and a lower ratio of exciton to vacancy peaks, indicating greater non-stoichiometry and lower crystallinity. Both samples have peaks associated with oxygen vacancies, whose change with humidity suggests that these vacancies are located in the surface layer.

**Figure 5:**
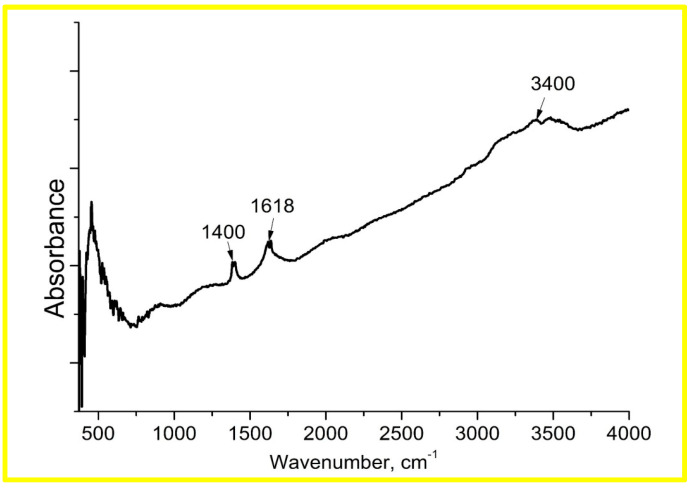
The difference in FTIR spectra of ZnO samples after prolonged exposure between wet and dry conditions at room temperature.

The FTIR spectra from Figure 5 show the effect of a wet atmosphere on the ZnO sample at room temperature. The curve on this graph represents the difference in spectrum between wet and dry conditions. The increase in intensity of the peaks at 1400 cm^−1^, 1618 cm^−1^ and 3400 cm^−1^ is observed after keeping the samples in a wet atmosphere. According to literature data, the molecularly adsorbed H_2_O on the ZnO (1010) surface is clearly identified by the characteristic scissoring mode at 1618 cm^−1^. Two peaks at 1400 and 3400 cm^−1^ are tentatively attributed to hydroxyl groups, as reported previously [43,44]. The broad peak at about 500 cm^−1^ is usually associated with intermolecular vibrations in water.

### 3.2. Effects of Wet Atmosphere on Electrical Characteristics of ZnO Nanorods Arrays

**Figure 6:**
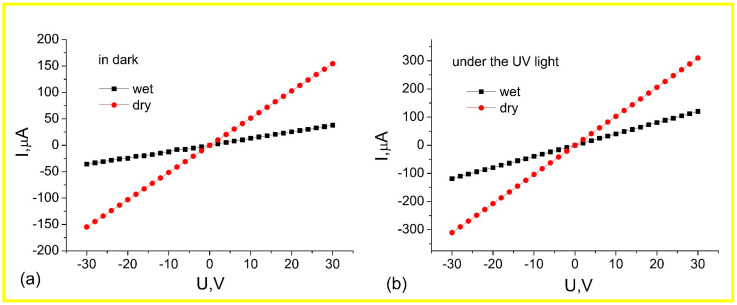
I-V characteristics in the dark (**a**) and under UV light (**b**) for the samples stored in dry and wet atmospheres.

**Figure 7:**
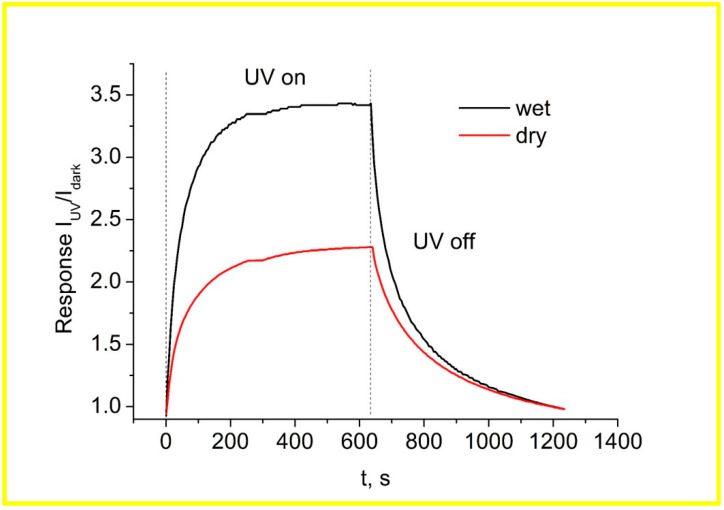
Time-dependent I_UV_/I_dark_ response of the ZnO nanorods UV detector at an applied voltage of 5 V for turning on and off UV irradiation at 370 nm for samples stored for a long period in a dry and wet atmosphere.

I-V curves measured on ZnO nanorod arrays in the dark and under UV irradiation for the samples stored in dry and wet atmospheres are shown in Figure 6. It can be seen that these dependencies are linear, i.e., the contacts are practically ohmic or their resistance is significantly lower than that of the nanorods [39]. The dark and UV resistances of the ZnO nanorod arrays are higher for samples stored in a wet atmosphere (Figure 7). The dark resistances were 1.9 × 10^5^ and 7.5 × 10^5^ Ohm for samples stored in dry and wet atmospheres, respectively. The UV resistances were 9.7 × 10^4^ and 2.4 × 10^5^ Ohm for samples stored in dry and wet atmospheres, respectively.

It was observed that the photocurrent of the sample stored in a dry atmosphere for 25 days increased rapidly from 3.6 × 10^−5^ to 8.8 × 10^−5^ A in about 235 s and then gradually saturated. When the UV light is turned off, the photocurrent decreases exponentially from 8.8 × 10^−5^ to 3.6 × 10^−5^ A in the first 390 s after the irradiation is turned off. The photocurrent of the sample stored in a wet atmosphere for 25 days increases rapidly from 6.37 × 10^−6^ to 2.43 × 10^−5^ A in about 170 s and then gradually saturates. After turning off the UV irradiation, the photocurrent decreased exponentially and returned to its original value within 320 s. In both cases, the rate of increase with irradiation is much faster than the rate of decrease when UV light is switched off.

It is obvious that after storing the samples in a wet atmosphere, the dark current and the photocurrent become significantly lower. Storage of the samples in a wet atmosphere for a long time leads to an improvement in the photoresponse time and sensitivity.

**Figure 8:**
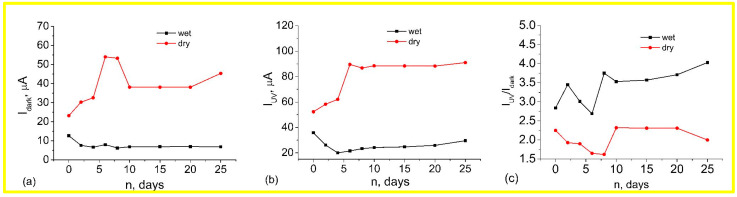
Dark current (**a**), photocurrent (**b**) and I_UV_/I_dark_ responses (**c**) from the storage over 25 days in the dry and wet atmosphere of ZnO nanorod arrays. The measurement error of the instruments is 1.1%.

The results of the measured dependencies of dark current, photocurrent, and UV response from the storage over 25 days in dry and wet atmospheres are shown in Figure 8. The measurement error was the sum of the power source and multimeter errors and was 1.1%. From this figure, it can be seen that for the samples stored in a humid atmosphere, there is a decrease in both the dark current and the photocurrent in the first few days. The characteristics then stabilize and show almost no change in the future. At the same time, the samples stored in a dry atmosphere show some increase in both currents throughout the time. As can be seen from Figure 8c, the sensitivity of the sample from the dry atmosphere almost does not change with time, whereas the sensitivity of the sample stored in a wet atmosphere increases significantly and then stabilizes at a higher level.

**Figure 9:**
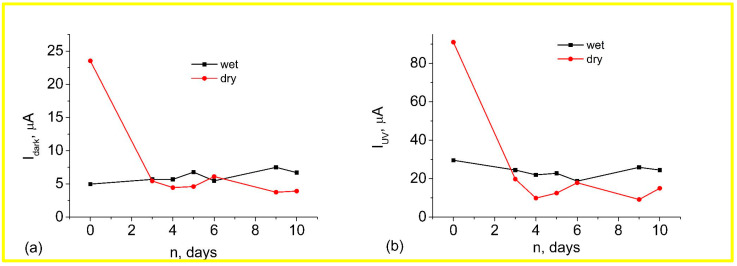
Dark current (**a**) and photocurrent (**b**) of ZnO nanorod arrays after 10 days of sample exchange. The measurement error of the instruments is 1.1%.

After the samples were exchanged, the samples stored in a dry atmosphere showed the same processes when placed in a wet atmosphere as the samples stored in a wet atmosphere. At the same time, the samples stored in a wet atmosphere showed practically no change in characteristics when placed in a dry atmosphere (Figure 9).

**Figure 10:**
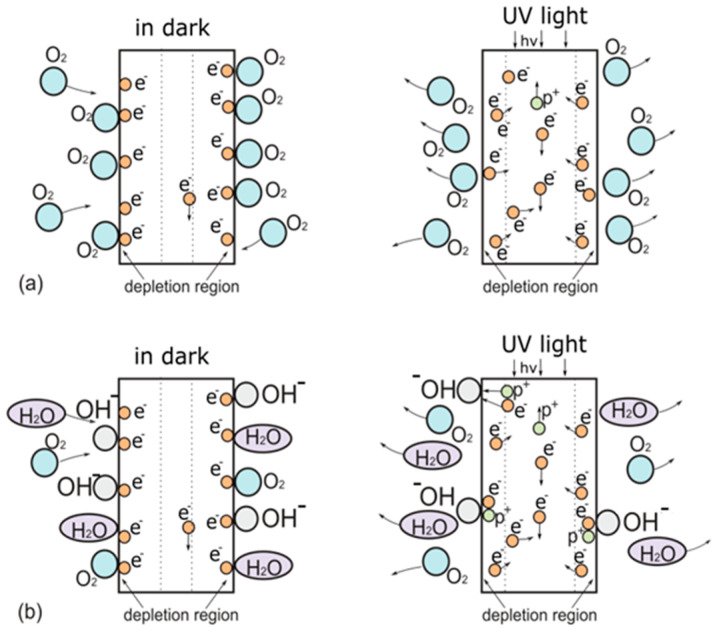
Schematic of the photoresponse process in ZnO nanorod arrays in dry (**a**) and wet (**b**) atmosphere.

To explain the results, we propose the following explanation of the surface effects of ZnO nanorod arrays in dry and wet atmospheres in the dark and under UV light irradiation (Figure 10). It is known that in a dry atmosphere, the photoresponse of ZnO nanorods is mainly determined by the adsorption and desorption of oxygen molecules on the surface of O_2_(g) + e^−^ → O_2_^−^ [30]. In the dark, oxygen molecules adsorb on the surface of ZnO nanorods and capture free electrons. As a result, the density of charge carriers and their mobility in the nanorods decreases, and a depleted region is formed. (Figure 10a). This process leads to a bending of the energy band near the surface [21]. The working surface area of nanorod arrays is significantly larger than that of films, so oxygen molecules significantly reduce the conductivity.

Under UV light irradiation, electron-hole pairs are generated and immediately contribute to the current. The resulting holes migrate to the surface and reduce oxygen to neutral molecules, which are desorbed from the surface of the nanorods. O_2_^−^ + h^+^ → O_2_(g). After turning on the UV radiation, the density of electrons becomes much higher than the density of holes, and their mobility increases. This leads to the almost complete disappearance of the depletion region, and consequently, the bending of the energy band decreases.

In a wet atmosphere, in addition to the adsorption of oxygen molecules, photosensitivity is also affected by the adsorption of water molecules on the surface. (Figure 10b). In the dark, water molecules partially replace adsorbed and ionized oxygen. The water adsorbed on the surface is a dimer of two water molecules, which can react with adsorbed oxygen by the reaction 2H_2_O(g) + O_2_ + 4e^−^ → 4OH^−^ [45]. OH^−^ hydroxyl groups form a stronger chemical bond with the zinc oxide surface, while undissociated water and oxygen molecules form a physical bond. This makes it easier for oxygen and water molecules to detach from the surface. Water is a dipole and can also act as an electron trap, increasing the width of the depletion region. This can also explain a decrease in CL intensity after storage in a wet atmosphere, as radiation recombination in the depletion region is suppressed. When the UV light is turned on, oxygen and water molecules are desorbed from the surface. The hydroxyl groups (OH^−^) remain on the crystallographic plane (100) and can capture both electrons and holes simultaneously, reducing the carrier density and increasing the depleted region, resulting in a lower observed photocurrent than for a sample in a dry atmosphere.

Thus, the changes that occur in the samples during storage in a wet environment are due to the formation of hydroxyl groups on the surface of the zinc oxide nanorods that are chemically bound to the zinc oxide. When UV light exposure is turned on, these groups are not desorbed, unlike the physically bound oxygen and water molecules. As a result, both the dark current and the irradiation current are reduced. At the same time, the dark current decreases more, leading to an increase in the relative change in current during irradiation and in sensitivity. When a sample that has been stored in a humid atmosphere for a long time is exposed to a dry atmosphere, chemically bound OH^−^ groups are also retained on the surface, which is also confirmed by FTIR spectral data. Therefore, the properties of such a sample do not change.

## 4. Conclusions

In summary, the effect of near 100% humidity on the sensing properties of arrays of ordered ZnO nanorods grown by CVD has been investigated. Qualitative studies show that the structure of the material is close to a single crystal. In order to understand the degradation mechanism, the study of the effect of humidity on the sensory properties of the samples was studied as a function of storage time. The increase in the sensitivity and response of ZnO nanorod arrays was observed after prolonged exposure to wet atmosphere. An important finding is that the expected increase in the resistance of a sample located in a wet atmosphere reaches saturation. This means that the sensor remains operational, and further exposure to a humid environment does not change its performance. An equally interesting result is a significant increase in the photoresponse of a sensor placed in a wet atmosphere. In addition to the practical use of this effect in real devices, the result has important scientific significance. In order to exclude the possibility that the samples initially had different properties, the samples were interchanged; the experiment showed that the results were reproducible. A model is proposed to explain the effects occurring in the sample, which is confirmed by FTIR spectral data.

Thus, it has been shown that UV sensors acquire stable characteristics that do not deteriorate subsequently, regardless of the level of humidity in the environment. The results obtained in this work on the study of the UV sensor characteristics of zinc oxide nanorod arrays can serve as a basis for the creation of cheap, easy-to-manufacture UV sensor devices intended for mass use.

## Data Availability

Data are contained within the article.
